# Polysaccharide-Based Hydrogel from Seeds of *Artemisia vulgaris*: Extraction Optimization by Box–Behnken Design, pH-Responsiveness, and Sustained Drug Release

**DOI:** 10.3390/gels9070525

**Published:** 2023-06-28

**Authors:** Muhammad Ajaz Hussain, Arshad Ali, Tariq G. Alsahli, Nadia Khan, Ahsan Sharif, Muhammad Tahir Haseeb, Omar Awad Alsaidan, Muhammad Tayyab, Syed Nasir Abbas Bukhari

**Affiliations:** 1 Centre for Organic Chemistry, School of Chemistry, University of the Punjab, Lahore 54590, Pakistan; ch.ahsan.chem@pu.edu.pk; 2 Institute of Chemistry, University of Sargodha, Sargodha 40100, Pakistan; arshadali04@yahoo.com (A.A.); arshirana207@gmail.com (N.K.); 3 Department of Pharmacology, College of Pharmacy, Jouf University, Sakaka 72388, Saudi Arabia; tgalsahli@ju.edu.sa; 4 College of Pharmacy, University of Sargodha, Sargodha 40100, Pakistan; mtahir212@yahoo.com; 5 Department of Pharmaceutics, College of Pharmacy, Jouf University, Sakaka 72388, Saudi Arabia; osaidan@ju.edu.sa; 6 Department of Pharmacy, Quaid-i-Azam University, Islamabad 45320, Pakistan; ph.tayyab@gmail.com; 7 Department of Pharmaceutical Chemistry, College of Pharmacy, Jouf University, Sakaka 72388, Saudi Arabia

**Keywords:** *Artemisia vulgaris* hydrogel, pH-responsiveness, response surface methodology, sustained release, Box–Behnken design

## Abstract

The current research work focuses on the extraction and optimization of the hydrogel (AVM) from the seeds of *Artemisia vulgaris* using Box–Behnken design-response surface methodology (BBD-RSM). The AVM was obtained through a hot water extraction process. The influence of different factors, including pH (U = 4 to 10), temperature (V = 25 to 110 °C), seed/water ratio, i.e., S/W ratio (W = 1/10 to 1/70 *w*/*v*), and seed/water contact time, i.e., S/W time (X = 1 to 12 h) on the yield of AVM was evaluated. The *p*-value for the analysis of variance (ANOVA) was found to be <0.001, indicating that the yield of AVM mainly depended on the abovementioned factors. The highest yield of AVM, i.e., 15.86%, was found at a pH of 7.12, temperature of 80.04 °C, S/W ratio of 1/33.24 *w*/*v*, and S/W time of 8.73 h according to Design-Expert Software. The study of the pH-responsive behavior of AVM in tablet form (formulation AVT3) revealed that AVM is a pH-responsive material with significantly high swelling at pH 7.4. However, less swelling was witnessed at pH 1.2. Moreover, AVM was found to be a sustained release material for esomeprazole at pH 7.4 for 12 h. The drug release from AVT3 was according to the super case-II transport mechanism and zero-order kinetics.

## 1. Introduction

The mucilaginous food polysaccharides/hydrogels of plant seed origin are biocompatible, non-toxic, and environmentally friendly [1]. They are commercially available at a low cost as compared to synthetic and semi-synthetic polymeric materials [2,3]. Such materials can absorb and retain large amounts of biological fluid without dissolving into them [4]. The structure of these materials is three-dimensional. Moreover, they have been extensively used in food industries as texture modifiers of different food items, gelling agents, and stabilizers in jellies as well as salad dressings. Additionally, they have also been utilized as pharmaceutical and industrial excipients for drug delivery systems (DDSs) and water purification before and after chemical modifications [5,6,7].

The seeds of the *A. vulgaris* (Syn: mugwort) is a new source of food hydrocolloid. Different parts of *A. vulgaris* have been used for the treatment of gynecological issues and gastrointestinal diseases for several centuries [8,9]. The seeds released super porous, biocompatible, non-toxic, and pH-responsive hydrogel, i.e., AVM has a wide range of pharmaceutical applications [10,11]. AVM is a chemically modifiable biomaterial due to the presence of different hydrophilic functional groups, such as –OH, –COOH, and –NH_2_, and appeared as an important material for different pharmaceutical, biomedical, and industrial applications [12]. However, the studies regarding the optimization of ideal extraction conditions in order to get the highest yield of AVM have not been reported so far to the best of our knowledge.

To extract the hydrogel from plant seeds, the aqueous extraction method has appeared as one of the most common extraction methods [13]. By varying the extraction conditions, the yield of the hydrogel extracted from the plant seeds has been observed. The extraction process has been reported to be significantly dependent on the pH, temperature, S/W ratio, and S/W time [14]. At high pH, temperature, S/W ratio, and S/W contact time, high energy is required for the extraction of hydrogels. Therefore, it is very important to optimize the ideal conditions to obtain a high yield of hydrogels through a suitable statistical design [15]. 

RSM is one of the most efficient statistical and mathematical tools for the optimization of yield whenever the hydrogel extraction process depends on multivariable factors [16]. It provides statistical (ANOVA) and graphical (2-dimensional contour as well as 3-dimensional response surface plots) procedures to study the authenticity of model design and the relationship between one or more responses [17,18]. Therefore, in this research work, we aimed to extract the AVM from the seeds of *A. vulgaris* using hot water extraction. After the collection of preliminarily extraction yield data for AVM, our main focus was to examine the effect of different parameters, i.e., pH (U), temperature (V), S/W ratio (W), and S/W time (X) on the yield (%Y) of AVM in terms of linear, squared, and quadratic functions. The AVM yield data will be verified statistically through BBD-RSM. Moreover, this study also aimed to find the pH-responsive sustained release potential of AVM for the delivery of esomeprazole after compression into a tablet dosage form. The compatibility between AVM and esomeprazole will be verified by recording their FTIR spectra. 

## 2. Results and Discussion

### 2.1. AVM Extraction 

**Figure 1:**
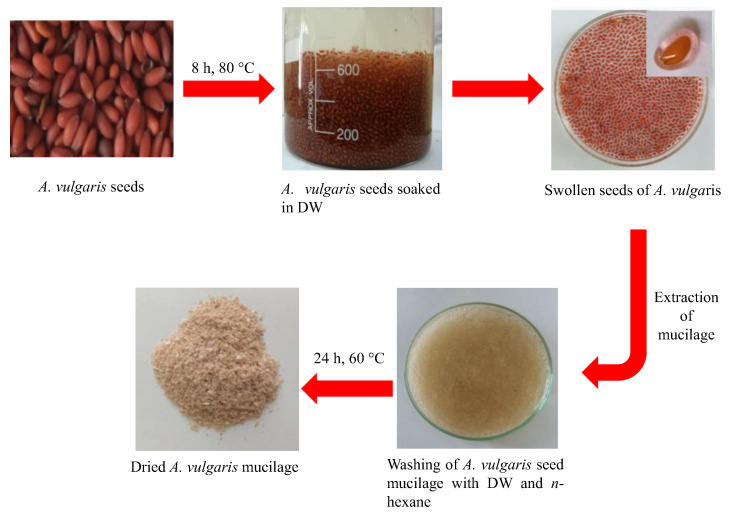
Scheme for the extraction of AVM from *A. vulgaris* seeds.

*A. vulgaris* seeds absorb water molecules upon soaking in deionized water (DW) due to the presence of micro-pores in them and due to the hydrophilic nature of AVM. The seeds swelled after absorbing DW and released the polysaccharide-based AVM. The AVM appeared colorless in dry powder form. The whole AVM extraction scheme is presented below (Figure 1).

### 2.2. Effect of Factors Affecting the AVM Yield

#### 2.2.1. pH

**Figure 2:**
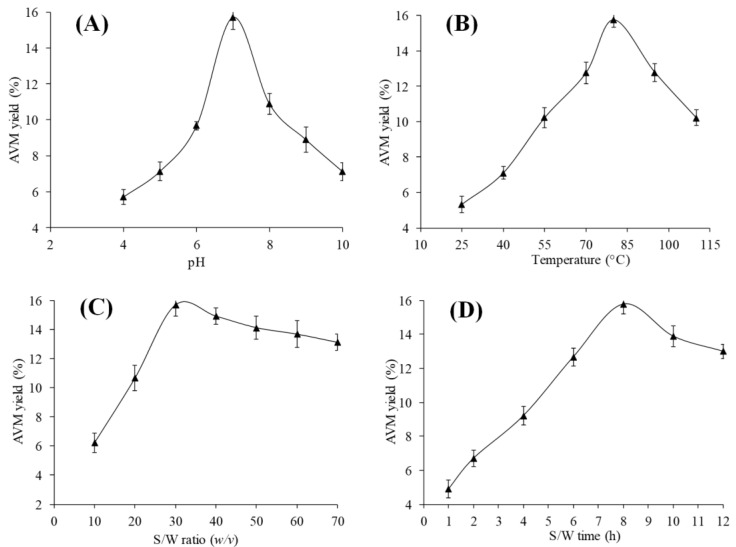
Plot illustrations showing the influence of pH (**A**), temperature (**B**), S/W ratio (**C**), and S/W time (**D**) on the AVM yield.

The influence of pH on the yield of AVM was studied by keeping a constant temperature of 80 °C, an S/W ratio of 1/30 *w*/*v*, and an S/W time of 8 h. A negligible amount of AVM yield, such as <5%, was obtained at pH ranging from 0 to 4 (yield data was not reported). Therefore, a pH ranging from 4 to 10 was selected to idealize the optimal pH (Figure 2A). At pH ranging from 4 to 6, the yield of AVM was found to increase up to 9.6%. However, a maximum yield of AVM = 15.71% was obtained at a pH of 7. After that, the yield of AVM was decreased to 7.1% at a pH of 10. The highest yield of AVM at a pH of 7 was due to the greater swelling of AVM at that pH [10]. The reduction in the yield of AVM at alkaline pH was due to its ability to dissolve in an alkaline medium [16]. Moreover, at alkaline pH of 10, comparatively less yield of AVM, i.e., 7.1%, was obtained than that of acidic pH of 6, i.e., 9.6%, which may be due to the hydrolysis of insoluble components of AVM to soluble components in alkaline pH [18]. Similar results have been reported by Golalikhani et al. for the optimization of ideal extraction conditions to obtain the highest extraction yield of *Descurainia sophia* from its seeds [19].

#### 2.2.2. Temperature

At pH of 7, the S/W ratio of 1/30 *w*/*v*, and the S/W time of 8 h, the effect of temperature on the yield of AVM was examined by changing the temperature from 25 to 110 °C. The yield of AVM was recorded as 5.3% at 25 °C. However, the yield of AVM was increased from 5.3 to 15.7% with the increase in temperature from 25 to 80 °C. At 80 °C, the highest yield of AVM, i.e., 15.7%, was recorded, and afterward, the yield of AVM was decreased to 10.2% at 110 °C (Figure 2B). Such an increase in the yield of AVM upon increasing the temperature was due to the reduction of the thickness between the seeds and hydrogel. Due to this, seeds released a high proportion of AVM. However, there may be a chance of polysaccharide degradation after 80 °C; therefore, the yield of AVM was found to decrease after 80 °C. A similar trend was observed by Nazir et al. [20] for the optimization of hydrogel extraction conditions from *Ocimum basilicum* seeds. This trend may corroborate the thermal degradation of the polysaccharides present in the hydrogel upon the increasing temperature up to an optimum level and led to a decrease in the yield of hydrogels [16].

#### 2.2.3. S/W Ratio

Keeping constant pH of 7, the temperature of 80 °C, and an S/W time of 8 h, the effect of varying S/W ratio, i.e., from 1/10 to 1/70 *w*/*v*, on the yield of AVM was examined. An increase in the yield of AVM was observed from 6.24 to 15.67% by increasing the S/W ratio from 1/10 to 1/30 *w*/*v*. This increase in the yield of AVM was due to the possible increase in the driving force of water molecules on the seeds, which pushes the AVM to come out from the seeds [21]. Moreover, the presence of high water contents makes the slurry to be less sticky and provided a more efficient extraction yield of AVM [22]. However, beyond the S/W ratio of 1/30 *w*/*v*, no more significant change in the AVM yield was observed, which indicated the establishment of dynamic equilibrium between water molecules and AVM [23] (Figure 2C). The yield of hydrogels from seeds of *Cydonia oblonga* [2] and *Salvia verbenaca* [19] was also found to increase with the increase in the S/W ratio. 

#### 2.2.4. S/W Time

In this process, the effect of S/W time on the yield AVM was studied by changing the S/W time from 1 to 12 h. The remaining extraction factors were kept constant at their pre-optimized levels. The yield of AVM was increased from 4.9 to 15.77% with the increase in the S/W time from 1 to 8 h. This is due to the presence of high water content in the extraction medium; water molecules enter the seed coat and ejected the maximum yield of AVM. However, after 8 h, no more changes in the yield of AVM were observed due to the establishment of dynamic equilibrium [18,24,25] (Figure 2D). These results also agreed with the reports of Hung and Li and Compos et al. on the hydrogel extraction from the seeds of *Basella alba* [26] and *Salvia hyspanica* [27], respectively. 

According to the aforementioned preliminarily experiments, the yield of AVM was found to be significantly influenced by pH, temperature, S/W ratio, and S/W time. The maximum AVM yield of 15.85% (15.85 g/100 g) was obtained at a pH of 7, a temperature of 80 °C, an S/W ratio of 1/30 *w*/*v*, and an S/W time of 8 h. Therefore, based on these preliminarily optimal conditions, three distinct levels for each independent factor were selected. The levels were: lowest (pH of 6; a temperature of 60 °C; an S/W ratio of 1/20 *w*/*v*, and S/W contact-time of 4 h), medium (pH of 7; a temperature of 80 °C; an S/W ratio of 1/30 *w*/*v*, and S/W time of 8 h), and highest (pH of 8, the temperature of 100 °C; the S/W ratio of 1/40 *w*/*v,* and S/W-time of 12 h). Subjected to these conditions, a BBD-RSM was designed using Design-Expert Software version 11.1.2.1 (Stat-Ease Inc., Minneapolis, MN, USA) (Table 1).

### 2.3. Response Surface Modeling

#### 2.3.1. Interpretation of ANOVA

The yield of AVM obtained from a series of 29 different experiments was found in the range of 3.45 to 15.75% (Table 1). The second-order quadratic equation fitted best to the experimental yield data. The obtained second-order quadratic equation for the BBD-RSM in this study is given below.

Extraction yield of AVM (Y%) = 15.48 + 1.13U + 0.5267V + 1.25W + 0.1325X −
6.25U^2^ − 1.91V^2^ − 0.8517W^2^ − 3.10X^2^ + 1.23UV − 1.31UW − 0.7375UX −
0.8625VW + 0.0000VX + 2.43WX
(1)

The results of ANOVA (Table 2) indicated that the *p*-value for ANOVA was <0.001. It means that the BBD-RSM fitted significantly well to the extraction yield data of AVM because the *p*-values for significant, highly significant, and super significant are <0.05, <0.01, and <0.001, respectively. Moreover, the ANOVA also explained the effect of tested factors on the yield of AVM in the form of linear, quadratic, and interaction terms based on *p*-values.

In the case of linear independent factors, the *p*-values were found to be <0.001 for pH, 0.0204 for temperature, <0.0001 for S/W ratio, and 0.5215 for S/W time. It shows that both pH and S/W ratio have a super significant effect, temperature has a significant effect, and S/W time has a non-significant effect on AVM yield (Table 2). Hence, the AVM yield related linearly to the pH, temperature, and S/W ratio and non-linearly to the S/W time.

The evaluation of the influence of independent factors in squared form (U^2^, V^2^, W^2^, X^2^) indicated that all the factors have a pronounced effect on the AVM yield because the *p*-values were found to be <0.001 in all cases. The effects of the interaction terms, i.e., S/W ratio and S/W time (WX) was found to be super significant, pH and S/W ratio (UW), and pH and temperature (UV) were found highly significant, temperature and S/W ratio (VW) was found significant, whereas temperature and S/W time (VX) and pH and S/W time (UX) and were found non-significant. The overall order was found to be VX (*p* = 1.0000) < UX (*p* = 0.0530) < VW (*p* = 0.0269) < UV (*p* = 0.0034) < UW (*p* = 0.0021) < WX (*p* = 0.0001) (Table 2).

The values of *R*^2^, *R*^2^_-adjusted_, and *R*^2^_-predicted_ were compared to assess the adequacy of BBD-RSM for the extraction yield data of AVM. The difference between *R*^2^ (0.9815) and *R*^2^_-adjusted_ (0.9630) was found to be 0.0185, and the difference between *R*^2^_-adjusted_ (0.9630) and *R*^2^_-predicted_ (0.9094) was found to be 0.0536 (Table 2). These negligible differences and the high value of *R*^2^ indicated that the designed model (BBD-RSM) was adequate and suitable for the AVM yield data [15].

A useful term for the assessment of the fitness level of any model with high precision in the results, reliability of the results, reproducibility of the results, and the adequacy of the model design is the measurement of coefficient of variance (%CV). If the %CV is less than 10%, then the corresponding model fits well with high precision and hence is highly reliable, whereas if the %CV is greater than 10%, then the corresponding model does not suitable due to low precision and reliability [27]. In the present study, the %CV was found to be 6.67% which is less than 10% (Table 2). Hence, demonstrated that the BBD-RSM was adequate and desirable for the identification of ideal conditions at which *A. vulgaris* seeds released a high yield of AVM.

The desirability of the model can also be determined in terms of signal/noise ratio by valuing adequate precision (ADP). Generally, there exists an inverse relationship between the values of ADP and signal/noise ratio and a direct relationship between the values of ADP and model desirability. It means that the greater the values of ADP lower will be the signal/noise ratio, and the greater will be the model desirability. Moreover, 4.0 is the normal value of ADP. If the value of ADP is >4.0, then the model is desirable, and vice versa [16]. In this present study, the ADP value was found to be 22.59, which is greater than the normal value (Table 2). This indicated that the BBD-RSM is desirable to optimize the ideal conditions for obtaining the highest AVM yield.

Pure errors and lack of fit are the two most important characteristics while describing whether a model fits well to the experimental yield data or not. In the case of a significant “lack of fit”, the model design cannot be used to analyze the experimental data, and the response predictor should be neglected. While in the case of a non-significant “lack of fit” the model can be successfully used to analyze the experimental data, and the response predictor cannot be neglected [18]. The value of the pure error for ANOVA for the optimization AVM yield was found to be 0.3497% with a non-significant lack of fit at a 95% confidence interval (Table 2). Therefore, the BBD-RSM is an appropriate model for the optimization of AVM yield.

#### 2.3.2. Elucidation of Response Surface-Plots

To analyze the effect of tested factors in binary form, the 3D-RS and 2D-C plots were obtained from BBD-RSM by keeping two factors constant at their optimized values and varying rests of the two factors. 

**Figure 3:**
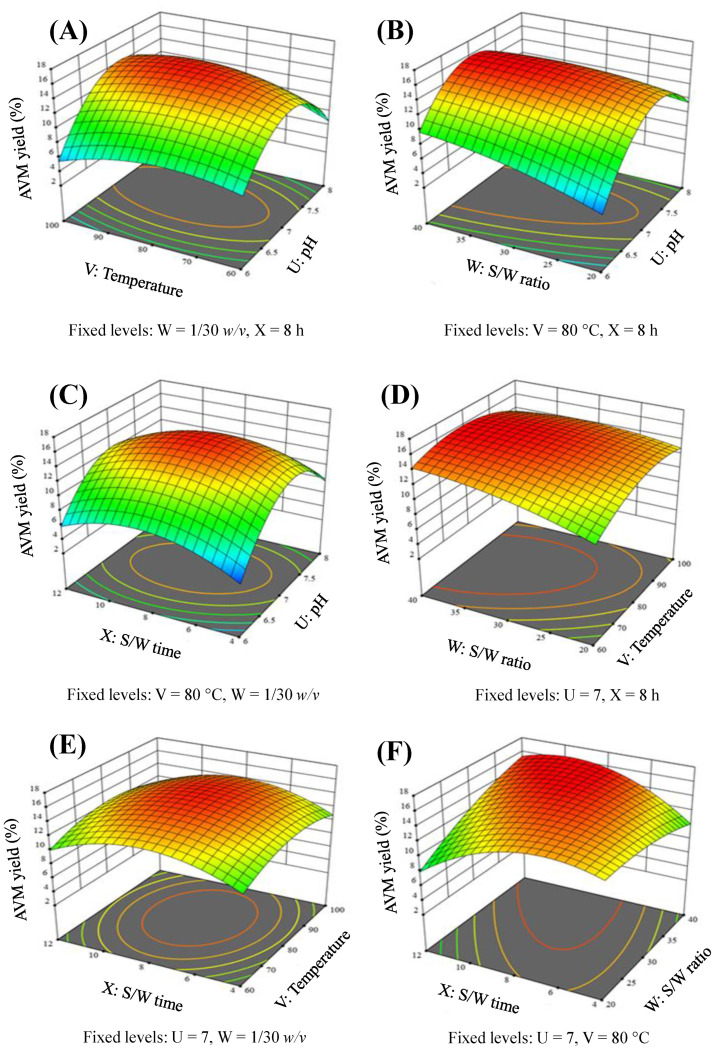
3D-RS plot illustrations indicating the influence of pH and temperature (**A**), pH and S/W ratio (**B**), pH and S/W time (**C**), temperature and S/W ratio (**D**), temperature and S/W time (**E**), and S/W ratio and S/W time (**F**) on the AVM yield.

**Figure 4:**
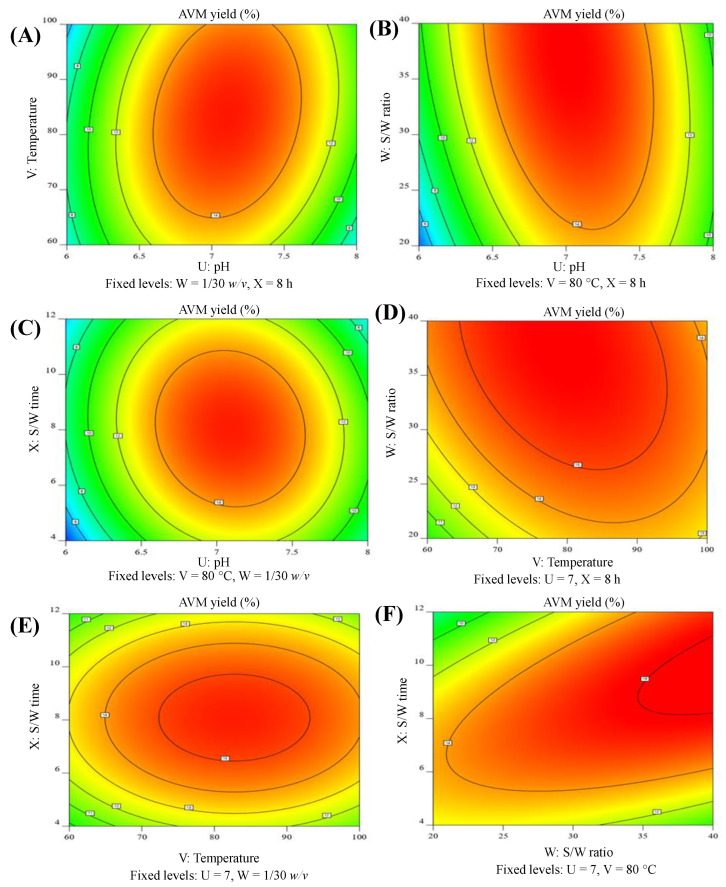
2D-C plot illustrations indicating the influence of pH and temperature (**A**), pH and S/W ratio (**B**), pH and S/W time (**C**), temperature and S/W ratio (**D**), temperature and S/W time (**E**), and S/W ratio and S/W time (**F**) on the AVM yield.

The 3D-RS and 2D-C figures indicating the interaction effect of pH and temperature are present in Figure 3A and Figure 4A. A considerable linear rise in the yield of AVM was seen by increasing the pH and temperature. The maximum AVM yield was found to be 15.55% at a pH of 7.04 and temperatures of 81.70 °C. However, the AVM yield declined to 10.30% at a pH of 7.99 and a temperature of 99.57 °C.

Figure 3B and Figure 4B is showing the influence of pH and S/W ratio in the combined form of 3D-RS and 2D-C plots, respectively. These figures clearly showed that the AVM yield increased linearly. The maximum AVM yield achieved was 15.85% at a pH of 6.98 and an S/W ratio of 1/37.108 *w*/*v*. However, beyond these threshold levels, the AVM yield tends to decrease.

At a fixed temperature of 80 °C and an S/W ratio of 1/30 *w*/*v*, the combined effects of the pH and the S/W time on the AVM yield were investigated. The obtained results are incorporated in Figure 3C (3D-RS plot) and Figure 4C (2D-C plot). These graphs showed that at low pH and S/W time, the AVM yield was negligible. However, with the increase in the pH and S/W time, the AVM yield also increased. The maximum AVM yield of 15.52% was obtained at a pH of 7.10 and an S/W time of 8.16 h. However, beyond these levels, the AVM yield started to decline sharply and reached a minimal level of 7.22% at a pH of 7.96 and seed/water contact time of 11.92 h.

The combined effect of temperature and the S/W ratio was evaluated at a fixed values of pH 7 and S/W time of 8 h (Figure 3D (3D-RS plot) and Figure 4D (2D-C plot)). Both of these factors significantly affected the yield of AVM. It was found that as the temperature increased from 60 to 80 °C and the S/W ratio increased from 1/20 to 1/30 *w*/*v*, the yield of AVM also increased. The highest yield of AVM, i.e., 15.46% was obtained at a temperature of 80.31 °C and a S/W ratio of 1/29.85 *w*/*v*. After that, the yield of AVM decreased slightly to 13.715% at a temperature of 99.71 °C and the S/W ratio of 1/39.80 *w*/*v*.

At pre-optimized pH of 7 and S/W ratio of 1/30 *w*/*v*, the quadratic effect of temperature and S/W time on the AVM yield was studied. Nearly a similar trend in the increase and decrease of the AVM yield was obtained as in the case of the combined effect of temperature and S/W ratio (Figure 3E (3D-RS plot) and Figure 4E (2D-C plot)). At the temperature of 81.85 °C and S/W time of 7.98 h, the highest AVM yield of 15.51% was obtained.

The dependency of the AVM yield on different S/W ratios and S/W time was also evaluated at a constant temperature of 80 °C and pH of 7. The findings were obtained in the form of a 3D-RS plot (Figure 3F) and a 2D-C (Figure 4F) plot. Plots showed that the AVM yield was low, i.e., 10.44%, at an S/W ratio of 1/39.79 *w*/*v* and S/W time of 4 h. However, after these levels, a linear relationship between these varying factors and the AVM yield was found. The seeds of *A. vulgaris* released 15.85% AVM at an S/W ratio of 1/39.41 *w*/*v* and an S/W time of 8.04 h.

In the 3D-RS plots (Figure 3A–F), the regions near 15% were bulged out and showed the optimal region of highest yield of AVM, whereas in the 2D-C plots (Figure 4A–F), the red areas marked with discrete round dark lines, indicating the regions of the optimum yield of AVM.

#### 2.3.3. Checking of Model Adequacy and Desirability

**Figure 5:**
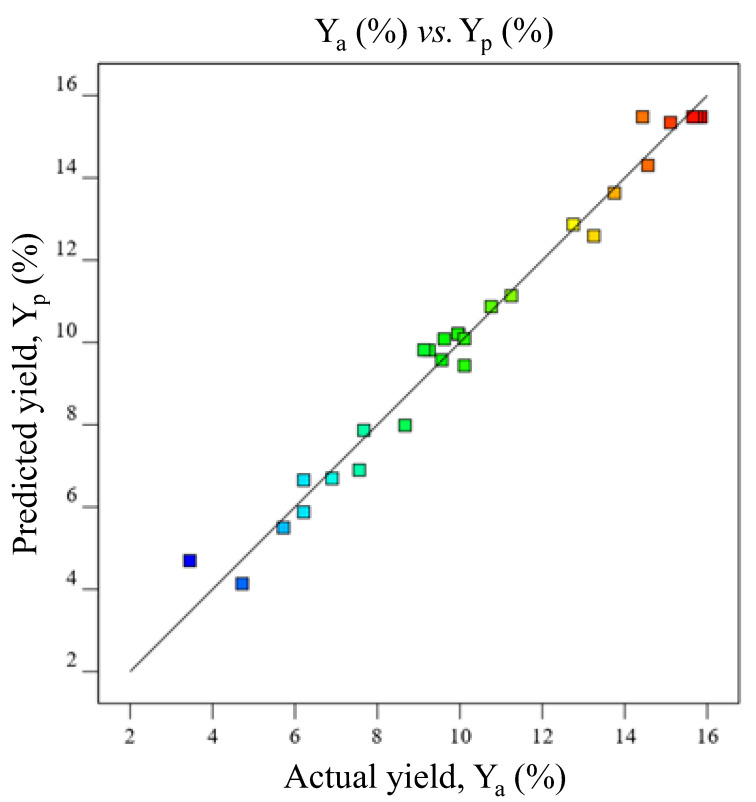
Scattered plot showing the comparison between Y_a_ and Y_p_ of AVM.

**Figure 6:**
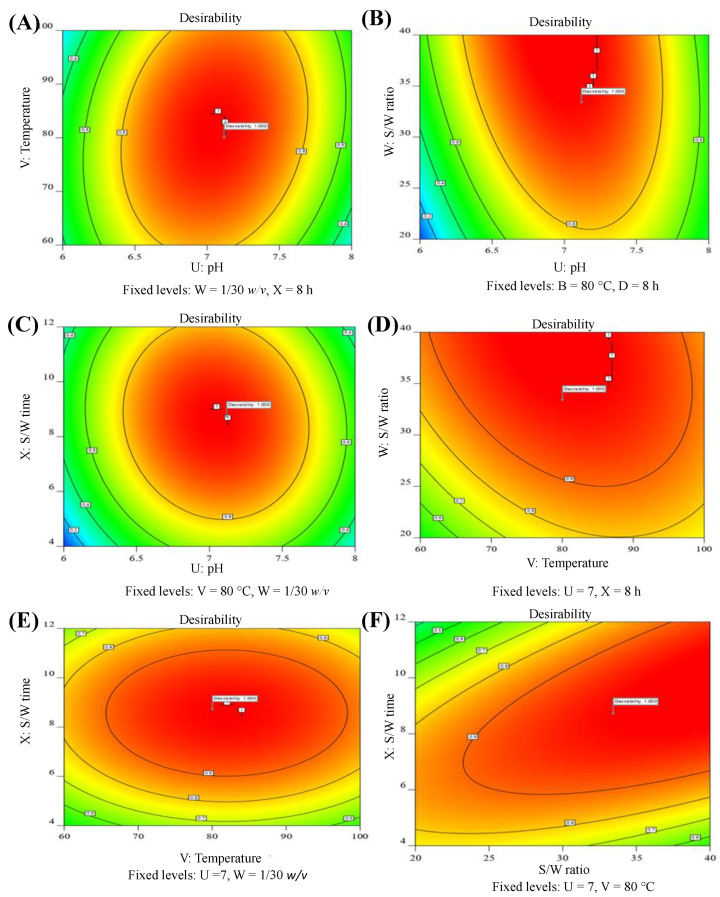
2D-desirability plot illustrations indicating the influence of pH and temperature (**A**), pH and S/W ratio (**B**), pH and S/W time (**C**), temperature and S/W ratio (**D**), temperature and S/W time (**E**), and S/W ratio and S/W time (**F**) on the AVM yield.

A scattered plot showing a comparison between Y_a_ and Y_p_ of AVM was obtained from the Design-Expert Software (Figure 5) to assess the adequacy of BBD-RSM for the AVM yield. The straight line of the plot represented the Y_p_ of AVM, whereas the randomly distributed scattered points on the straight line represented the Y_p_ of AVM. The points representing the Y_a_ were omitted to avoid a discrepancy between the Y_a_ and Y_p_ because an improper model could lead to an incorrect evaluation of yields. No significant difference between Y_a_ and Y_p_ was seen from the plots (Figure 4 and Table 1). Figure 6A–F shows the 2D-desirability plots. The desirability for each study of the combined effect of tested factors was found to be 1. Hence, it can be stated that the developed quadratic model and BBD-RSM were adequate and desirable for the optimization of ideal conditions to maximize the AVM yield.

#### 2.3.4. Optimum Conditions

**Figure 7:**
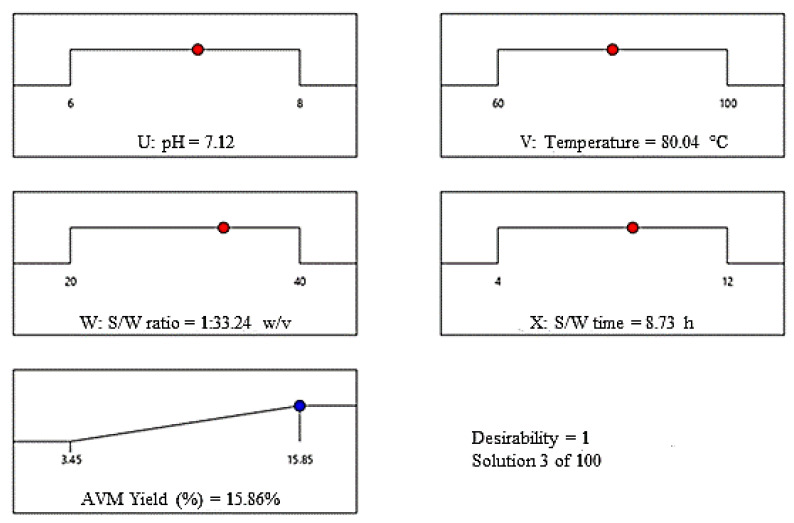
Optimization cycles for the extraction optimization of AVM.

According to the AVM yield results obtained from Design-Expert Software, the seeds of *A. vulgaris* released maximum amount of AVM, i.e., 15.86% at a pH of 7.12, a temperature of 80.04 °C, an S/W ratio of 1/33.24 *w*/*v*, and S/W time of 8.73 h (Figure 7). The red circle in the Figure 7 indicated these conditions. This optimized yield of AVM and optimal conditions are very close to the experimental yield of AVM, i.e., 15.85% experimental conditions, i.e., pH of 7, the temperature of 80 °C, S/W ratio of 1/30 *w*/*v*, and S/W time of 8 h (Table 1, run 21). Moreover, the AVM yield obtained from the scattered plots between Y_a_ and Y_p_ was also found in close agreement with the above ones under similar extraction conditions (Figure 5). Therefore, the extraction conditions presented in Table 2 at run no. 21 are the best conditions to obtain the highest AVM yield from *A. vulgaris* seeds.

#### 2.3.5. Comparison of AVM Yield with the Yield of Already Reported Hydrogels

The AVM yield was found to be greater as compared to the yield of already reported hydrogels (see Table 3). This indicated that the AVM could be an important future commercial gum for pharmaceutical and industrial applications after exploring more of its structural features. 

### 2.4. AVM as a Sustained Release Material

#### 2.4.1. Compatibility Studies

**Figure 8:**
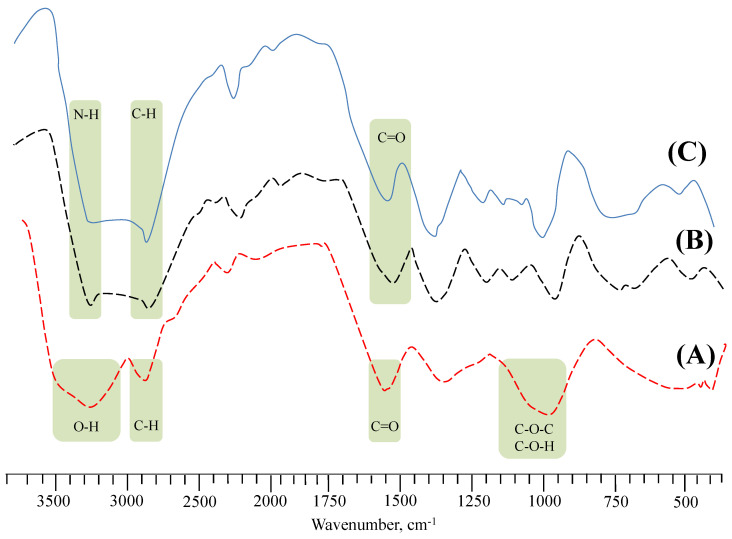
FTIR spectra of AVM (**A**), esomeprazole (**B**), and AVT3 (**C**).

Compatibility studies among the ingredients of the tablet formulations were performed through FTIR analysis (Figure 8). In the FTIR spectrum of AVM (Figure 8A), broadband appeared around 3100–3500 cm^−1^, indicating the presence of –OH groups [30]. The existence of COC glycosidic linkage and COH stretching is indicated through a band between 1000–1200 cm^−1^. In the FTIR spectrum of esomeprazole (Figure 8B), the band at 3415 and 2946 cm^−1^ corresponded to the –N–H and C–H groups, respectively [31]. Moreover, the band at 1589 cm^−1^ corresponded to the –C=O groups. The characteristic bands of AVM and esomeprazole can be observed in their physical mixture, indicating the absence of any physical and chemical interaction between them.

#### 2.4.2. pH-Responsive Swelling

**Figure 9:**
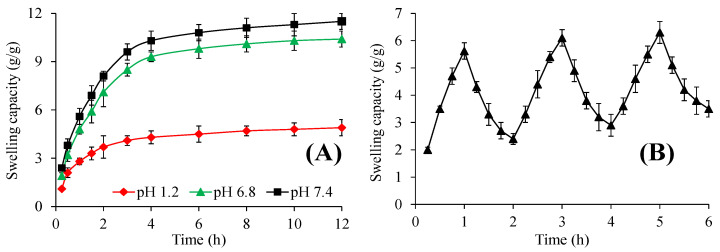
pH-responsive swelling behavior of AVT3 (**A**) and pH-responsive swelling/deswelling profile of AVT3 (**B**).

The pH-responsive swelling of AVT3 was evaluated at different pH, and the obtained results are incorporated in Figure 9A. The AVT3 swelled extremely well at pH 6.8 and 7.4. Minor swelling of AVT3 was observed at pH 1.2. The order of swelling of AVT3 was pH 7.4 > pH 6.8 > pH 1.2. A possible explanation for this behavior is that at pH 7.4, the carboxylic acid (–COOH) present on the polymeric backbone of AVT3 gets deprotonated due to the basic environment of pH 7.4 and ionized to carboxylate (–COO^−^) ions. As a result, it offers anion-anion, i.e., –COO^−^ and –COO^−^ electrostatic repulsions, due to which the swelling medium penetrated the polymeric networks of AVT3 and it swelled [32]. However, at pH 1.2, –COOH of AVM gets protonated due to the presence of acidic groups, and the attractive interactions between the polymeric chains of AVT3 became dominant. Consequently, the AVT3 swelled to a small extent only.

#### 2.4.3. pH-Responsive Swelling/Deswelling

The formulation AVT3 of AVM showed rapid swelling at pH 7.4, and it quickly deswelled after being transferred to the buffer of pH 1.2. This is because –COOH groups of AVM begin to change into –COO^−^ form at pH 7.4, and –COO^−^–COO^−^ repulsive forces operated between the alike –COO^−^ groups. As a result, the inter- and intra-molecular hydrogen bonds that were present at the polymeric backbone of AVT3 were destroyed, and AVT3 swelled abruptly. At pH 1.2, the –COO^−^ groups reverted to the protonated form, i.e., –COOH and AVM regained their intra- and intermolecular hydrogen bonding, which is responsible for its shrinking. The findings of the swelling/deswelling experiments of AVT3 (three cycles) described the reproducibility of the results (Figure 9B). Therefore, the AVT3 exhibited pH-responsive swelling/deswelling behavior.

#### 2.4.4. Esomeprazole Release and Release Mechanism

**Figure 10:**
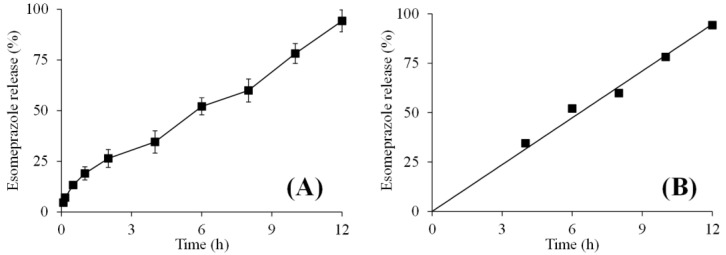
Cumulative release of esomeprazole from AVT3 at pH 6.8 (**A**) and zero-order kinetics of esomeprazole release from AVT3 at pH 6.8 (**B**).

The examination of esomeprazole release from AVT3 at pH 6.8 revealed a sustained-release pattern for 12 h. The esomeprazole was released according to the zero-order release kinetics (Figure 10A,B; Table 4). Korsmeyer–Peppas model was applied to study the mechanism of esomeprazole release. The values of *R*^2^ and *n* appeared as 0.9979 and 0.471, respectively, and indicated the non-Fickian diffusion mechanism of esomeprazole release (Table 4) [33,34,35].

## 3. Conclusions

The RSM-BBD was found to be a valuable statistical and graphical model for the optimization of extraction conditions to obtain the maximum yield of AVM from *A. vulgaris* seeds. The extraction yield data fitted well to the second-order quadratic equation. ANOVA for BBD-RSM in the current study exhibited a non-significant lack of fit with a high value of *R^2^*. The yield of AVM was mainly dependent on the pH of the extraction medium. The optimized conditions to achieve the maximum yield, i.e., 15.85% of AVM, were a pH of 7.11, a temperature of 80.03 °C, an S/W ratio of 1/33.24 *w*/*v*, and an S/W time of 8.73 h using Design-Expert Software. The studies on pH-responsive swelling, swelling/deswelling, and esomeprazole release from AVM-based tablets showed that AVM is a pH-responsive material with the potential for a sustained-release drug delivery system.

## 4. Materials and Methods

### 4.1. Materials

*A. vulgaris* seeds were purchased from Seed Needs, LLC, Lahore, Pakistan. Before extracting the AVM from *A. vulgaris* seeds, the cleaning steps, including de-husking, splitting, de-dusting manually, and washing with DW, were carried out to remove filthy materials from the seeds. Analytical-grade chemicals were procured from Riedel-de Haën, Germany. The buffers of pH 1.2, 6.8, and 7.4 were prepared using a standard protocol as described in the United States Pharmacopeia (USP 34-NF 29). The model drug, esomeprazole (European Pharmacopoeia (Ph. Eur.) standard), was gifted by Dyson Research Laboratories, Lahore. The whole experimental work was performed using DW.

### 4.2. Methods 

#### 4.2.1. AVM Extraction

The clean seeds of *A. vulgaris* were soaked in the DW using an S/W ratio of 1/30 *w*/*v* at 80 °C for 8 h. The hydrogel, i.e., AVM, released from seeds was separated by rubbing the swollen seeds on a nylon sieve with the help of a spatula. The non-polar and polar contaminants were removed by purifying the AVM with *n*-hexane and DW, respectively. The purified AVM was dried in a hot vacuum oven for 48 h at 60 °C. The dried AVM was milled and passed through mesh no. 60 to make finely divided powder. Finally, the AVM was stored in an air-tight jar until further use in the experimental work.

#### 4.2.2. Determination of Experimental Yield

The experimental yield of AVM was determined using Equation (2) [15]:

(2) $$Experimental\;yield\;of\;\mathrm{AVM}\;\left(\%\right)=\frac{Dry\;weight\;of\;\;\mathrm{AVM}\;}{\begin{array}{c} Weight\;of\;A.\;\;vulgaris\;seeds\\ taken\;for\;mucilage\;extraction\; \end{array}}\times 100$$

#### 4.2.3. Design of Experimentation and Statistical Validation 

Before designing a model for optimizing the best conditions to acquire the maximum yield of AVM and to check the effect of a single independent factor, i.e., pH (U = 1 to 10), temperature (V = 25 to 110 °C), S/W ratio (W = 10/70 *w*/*v*), and S/W time (X = 1 to 12 h) on the extraction yield of AVM, some basic experimental studies were conducted. Based on these experimental studies, three levels designated as lowest (−1), medium (0), and highest (+1) were selected for each factor so that the effect of these factors on the yield of AVM in binary form could be monitored. The levels were: U (lowest = 6, medium = 7, and highest = 8), V (lowest = 60 °C, medium = 80 °C, and highest = 100 °C), W (lowest = 1/20 *w*/*v*, medium = 1/30 *w*/*v*, and highest 1/40 *w*/*v*), and X (lowest = 4 h, medium = 8 h, and highest = 12 h). The experimental setup consisting of 29 experiments was designed according to the BBD-RSM using Design-Expert Software. The statistical significance of all the tested factors was confirmed from the regression (ANOVA), and graphics (2D-C and 3D-RS) plots for the experimental yield of AVM.

In addition to the linear as well as quadratic effects, the effect of composite interactions on the yield of AVM was also studied while evaluating the model equation. Therefore, the mean values of the experimental yield data were fitted into a second-polynomial equation to predict the yield and statistical significance of the BBD-RSM. The linearized (general form) second-order equation is represented in Equation (3):

Extraction yield of AVM (*Y*%) = λ_o_ + λ_1_U + λ_2_V + λ_3_W + λ_4_X + λ_11_U^2^ +
λ_22_V^2^ + λ_33_W^2^ + λ_44_X^2^ + λ_1_λ_2_UV + λ_1_λ_3_UW + λ_1_λ_4_UX + λ_2_λ_3_VW + λ_2_
λ_4_VX + λ_3_λ_4_WX + *E_i_* (3)

where *Y* indicated the yield of AVM in percentage and the terms U, V, W, and X has already been described above. λ_o_ represented the intercept, λ_1_, λ_2_, λ_3_, and λ_4_, were the coefficients of linearity, λ_11_, λ_22_, λ_33_, and λ_44_ were the squared coefficients, and λ_1_λ_2_, λ_1_λ_3_, λ_1_λ_4_, λ_2_λ_3_, λ_2_λ_4_, and λ_3_λ_4_ were the interaction coefficients. The *E_i_* represented the error function in the designed model (if any).

The “fitness of good” of the second-order polynomial model equation and ANOVA to the extraction yield data of AVM was evaluated by noting the values of DF, *p*-, *F*-, *R^2^*, *R*^2^_-predicted_, and *R*^2^_-adjusted_. Moreover, the suitability of BBD-RSM to the extraction yield data of AVM was also estimated in terms of %CV, SSE, ADP, the PRESS, and lack of fit. The 3D-RS and 2D-C plots were obtained to see the connection between the yield of AVM and tested factors and to locate the regions of optimum experimental values of the tested factors and the model desirability. A scattered plot between the Y_a_ and Y_p_ of AVM was drawn to check the adequacy of BBD-RSM for extraction yield data of AVM.

#### 4.2.4. AVM as a Sustained Release Material

##### Preparation of AVM-Based Tablets

Three tablets of AVM, i.e., AVT1, AVT2, and AVT3, were prepared through a wet granulation method by changing the concentration of AVM in each [8,10]. AVM and esomeprazole were physically mixed in a pestle–mortar, followed by the addition of a 5% (*w*/*v*) solution of polyvinyl pyrrolidone (PVP) in isopropyl alcohol and added to the mixture of AVM and esomeprazole to moisten it. This mixture was homogenized, granulated, sieved through mesh no. 12, ground, dried at 40 °C for 8 h, and sieved again by passing through mesh no. 20. The granules were mixed with 10 mg of Mg-stearate and pressed on a rotary press fitted with an 11 mm flat surface punch. The thickness of each tablet was maintained between 6–9 kg/cm^2^. The composition of each tablet is mentioned in Table 5.

##### Compatibility Study

The Fourier transform infrared (FTIR, KBr pellets, 4000–500 cm^−1^) spectra of AVM, esomeprazole, and AVT3 were recorded on IR Prestige-21 (Shimadzu, Tokyo, Japan) to evaluate any possible interaction between them.

##### pH-Responsive Swelling

The tea bag method [36] was used to study the pH-responsive swelling properties of AV3. In a typical procedure, three AVT3 tablets (400 mg each) were packed in already-weighed tea bags and hung in the beakers containing buffers of pH 1.2, 6.8, and 7.4. The beakers were placed under shaking at 25 °C and stirred continuously. The tea bags from each buffer were taken out periodically, blotted to drain excessive buffer, and weighed. The swelling capacity (g/g) of AVT3 in each buffer was determined by applying Equation (4). The swelling studies of AVT3 were conducted in triplicate and reported the average values.

(4) Swelling capacitygg=Ws−We−WiWi

where *W_s_* (*g*) is the weight of tea bag having swollen AVT3, *W_e_* (*g*) indicated the weight of an empty wet tea bag, and *W_i_* (*g*) denotes the initial weight of AVT3. 

##### pH-Responsive Swelling/Deswelling

The swelling/deswelling capability of AVT3 was studied in the swelling medium (pH 7.4) and deswelling medium (pH 1.2). The AVT3 was packed in an already-weighed tea bag and shifted to a beaker containing a buffer of pH 7.4. The swelling of AV3 was noted for 1 h using Equation (4), and then the same tea bag was placed into a beaker containing a buffer of pH 1.2, and the swelling/deswelling capacity of AVT3 was noted using Equation (4). Three swelling/deswelling cycles were recorded thrice, and the means are reported here.

##### In Vitro Esomeprazole Release

The in vitro release study of the esomeprazole from AVT3 was conducted on USP Dissolution Apparatus II at pH 6.8 for a time period of 12 h. A dissolution vessel was filled with 900 mL of the buffer of pH 7.4. The temperature of the apparatus was maintained at 37 ± 0.5 °C. The rotational speed of the apparatus was maintained at 100 rpm. 10 mL of the dissolution sample was taken out from the dissolving medium periodically and filtered. The filtered sample was diluted with the same dissolution medium, and absorbance was noted at 285 nm on a UV-Vis spectrophotometer (UV-1600 Shimadzu, Duisburg, Germany). The level/volume of the dissolution medium was maintained to its inherent by adding the same dissolution medium. The esomeprazole release study was conducted in triplicate, and the average is reported here.

##### Kinetics and Mechanism of Esomeprazole Release

The esomeprazole release data were fitted to the zero-order kinetic model (Equation (5)) [37] and the Korsmeyer–Peppas model (Equation (6)) [33] to examine the rate and the mechanism through which the esomeprazole released from AVT3, respectively.

(5) $$Q_{t}=K_{0}t$$

(6) $$\frac{M_{t}}{M_{\infty }}=K_{p}t^{n}$$

where *Q_t_* showed the quantity of esomeprazole released at time *t*, *M_t_*/*M_∞_* indicated the amount of esomeprazole released at any time *t*, *K*_0_ is the rate constant for the zero-order kinetic model, *K_p_* represented the rate constant for the Korsmeyer-Peppas model, and *n* is the diffusion exponent. The value of *n* gives information regarding the mechanism of esomeprazole release from AVT3. The value of *n* ≤ 0.45 is for Fickian diffusion, 0.45–0.89 for non-Fickian diffusion, *n* = 0.89 for case-II transport, and *n* > 0.89 for super case-II transport [34,35].

## Figures and Tables

**Table 1:** BBD-RSM and the comparison between experimental (actual, Y_a_) and calculated (predicted, Y_p_) yields (%) of AVM.

Experiments	Tested Factors				Responses/Yields (%)	
	U	V (°C)	W (*w*/*v*)	X (h)	Y_a_	Y_p_
1	7	80	30	8	15.75	15.48
2	8	80	30	12	6.21	6.65
3	8	80	20	8	9.56	9.57
4	7	100	30	12	11.25	11.13
5	8	80	30	4	7.67	7.86
6	8	60	30	8	6.9	6.69
7	7	100	30	4	10.76	10.87
8	7	80	40	4	9.95	10.21
9	6	80	20	8	3.45	4.69
10	6	80	30	12	6.21	5.88
11	7	60	30	12	9.62	10.08
12	7	80	30	8	15.65	15.48
13	7	100	20	8	12.75	12.87
14	7	60	30	4	9.13	9.82
15	7	60	20	8	10.11	10.09
16	6	60	30	8	7.56	6.90
17	7	80	20	12	8.67	7.98
18	7	100	40	8	13.75	13.63
19	7	80	20	4	13.25	12.59
20	7	80	30	8	14.43	15.48
21	7	80	30	8	15.85	15.48
22	8	100	30	8	9.97	10.20
23	8	80	40	8	10.11	9.44
24	7	80	40	12	15.11	15.34
25	6	80	30	4	4.72	4.14
26	6	80	40	8	9.25	9.81
27	7	80	30	8	15.72	15.48
28	7	60	40	8	14.56	14.30
29	6	100	30	8	5.72	5.50

**Table 2:** ANOVA showing the results of the BBD-RSM for the optimized extraction conditions for AVM yield.

Source	SSE	DF	Mean	*F*-Values	*p*-Values
Model	361.82	14	25.84	53.05	<0.0001 ^ss^
Linear terms					
U-pH	15.21	1	15.21	31.22	<0.0001 ^ss^
V-Temperature (°C)	3.33	1	3.33	6.83	0.0204 ^s^
W-S/W ratio (*w*/*v*)	18.60	1	18.60	38.18	<0.0001 ^ss^
X-S/W time (h)	0.2107	1	0.2107	0.4325	0.5215 ^ns^
Quadratic terms					
U^2^	253.41	1	253.41	520.18	<0.0001 ^ss^
V^2^	23.58	1	23.58	48.40	<0.0001 ^ss^
W^2^	4.70	1	4.70	9.66	0.0077 ^hs^
X^2^	62.25	1	62.25	127.78	<0.0001 ^ss^
Interaction terms					
UV	6.03	1	6.03	12.37	0.0034 ^hs^
UW	6.89	1	6.89	14.14	0.0021 ^hs^
UX	2.18	1	2.18	4.47	0.0530 ^ns^
VW	2.98	1	2.98	6.11	0.0269 ^s^
VX	0.0000	1	0.0000	0.0000	1.0000 ^ns^
WX	23.72	1	23.72	48.68	<0.0001 ^ss^
Residual	6.82	14	0.4872		
Cor total	368.64	28			
Lack of fit	5.42	10	0.5421	1.55	0.3572 ^ns^
Pure error	1.40	4	0.3497		
*R*^2^ = 0.9815; *R*^2^_-adjusted_ = 0.9630; *R*^2^*_-_*_predicted_ = 0.9094; ±SD = 0.6980; %CV = 6.67%; Mean = 10.47; ADP = 22.59; Predicted error sum of square (PRESS) = 33.41					

SSE: Sum square error; DF: Degrees of freedom; ^s^ Significant (*p* < 0.05); ^hs^ Highly significant (*p* < 0.01); ^ss^ Super significant (*p* < 0.001); ^ns^ Non-significant.

**Table 3:** Comparison of AVM yield with other naturally occurring polysaccharide-based hydrogels.

Botanical Name of Plants	Common Name of Plants	Source of Hydrogel	Yield (%)	References
*Durio zibethinus*	Durian	Seeds	1.2	[28]
*Tiliacora triandra*	Yanang gum	Leaves	4.54	[29]
*Salvia hispanica*	Chia	Seeds	4.95	[27]
*Lepidium perfoliatum*	Cress	seeds	6.46	[25]
*Salvia spinosa*	Kannocha	Seeds	7.35	[16]
*Descurainia sophia*	Flixweed	seeds	10.45	[19]
*Mimosa pudica*	Touch-me-not	Seeds	10.66	[18]
*Artemisia vulgaris*	Mugwort	Seeds	15.86 (Highest)	Present study

**Table 4:** Kinetic data of esomeprazole release.

Tablet	Zero-Order Kinetic Model		Korsmeyer-Peppas Model		
AVT3	*R*^2^ 0.9942	*K*_0_ 7.97	*R* ^2^	*K*_*KP*_	*n*
			0.9979	18.3780	0.471

**Table 5:** Composition of tablets based on AVM and esomeprazole.

Composition of the Tablets	AVT1	AVT2	AVT3
AVM	150	200	250
Esomeprazole	80	80	80
Tragacanth gum	60	60	60
MC-cellulose	100	50	-
Mg-stearate	10	10	10
Total weight	400	400	400

## Data Availability

Not Available.

## Abbreviations

AVM (*Artemisia vulgaris* mucilage), BBD-RSM (Box–Behnken design-response surface methodology), ANOVA (Analysis of variance), DW (Deionized water), SSE (Sum square error), DF (Degrees of freedom), PRESS (Predicted error sum of square), ADP (Adequate precision), CV (Coefficient of variance), USP-NF (United States Pharmacopeia-National Formulary).
